# Prevalence and risk factors of asymptomatic *Plasmodium* spp. infection in the military population of the Colombian National Army

**DOI:** 10.1371/journal.pntd.0014441

**Published:** 2026-07-02

**Authors:** Carolina Oliveros, Camilo A. Correa-Cárdenas, Lorena I. Orjuela, Lorena Albarracin, Elizabeth K. Márquez, María T. Alvarado, Julie Pérez, Diego Chacón, Frank De Los Santos Ortíz, Yanira Romero, Pilar Ruiz-Jara, Vanessa Herrera-Jiménez, Carlos D. Daza, Maria Clara Duque, Yohana Rivera-Rincón, Claudia Méndez, Omar Cantillo-Barraza, Luz H. Patiño, Juan David Ramírez, Zulma M. Cucunubá

**Affiliations:** 1 Grupo de Investigación en Enfermedades Tropicales del Ejército (GINETEJ), Laboratorio de Referencia e Investigación, Dirección de Sanidad Ejército, Bogotá, Colombia; 2 Facultad de Ciencias, Pontificia Universidad Javeriana, Bogotá, Colombia; 3 Department of Clinical Epidemiology and Biostatistics, Facultad de Medicina, Pontificia Universidad Javeriana, Bogotá, Colombia; 4 Centro de Investigaciones en Microbiología y Biotecnología – UR (CIMBIUR), School of Sciences and Engineering, Universidad del Rosario, Bogotá, Colombia; 5 Center for Global Health and Interdisciplinary Research, USF Genomics Program, Department of Global, Environmental and Genomic Health Sciences, College of Public Health, University of South Florida, Tampa, Florida, United States of America; Advanced Centre for Chronic and Rare Diseases, INDIA

## Abstract

**Background:**

Asymptomatic malaria plays a critical role in sustaining transmission in endemic regions, yet its magnitude and determinants remain insufficiently characterized in military populations frequently exposed during field operations. This study sought to estimate the prevalence of asymptomatic *Plasmodium* spp. infection and identify associated risk factors among Colombian military personnel deployed in high-endemicity areas in 2022.

**Methodology/principal findings:**

A cross-sectional survey was conducted in four departments with the highest malaria transmission (Antioquia, Chocó, Córdoba, and Nariño). A total of 773 participants underwent thick blood smear microscopy, rapid diagnostic testing (RDT), conventional PCR, and real-time PCR. The prevalence of asymptomatic infection detected by conventional PCR/qPCR was 2.59%, with the highest municipal rates observed in El Bagre and Carepa (Antioquia), followed by Tumaco, Quibdó, and Tierralta. *P. falciparum* accounted for most infections (60%), followed by *P. vivax* (25%) and mixed infections (15%). qPCR demonstrated the greatest diagnostic sensitivity. Statistical analyses identified frequency of bed net use, number of lifetime and recent malaria episodes, department of origin, department and duration of patrol, number of patrol sites, and age as the main associated risk factors.

**Conclusions/significance:**

These findings highlight the relevance of asymptomatic *Plasmodium* spp. infections among Colombian military personnel and underscore the need to integrate their detection into routine malaria surveillance. Strengthening identification of low-density infections in highly exposed populations may contribute to reducing transmission, improving clinical management, and enhancing operational readiness in endemic areas.

## Author summary

Asymptomatic malaria infections occur when people carry the malaria parasite without showing symptoms. These silent infections are difficult to detect with routine diagnostic methods and can maintain transmission in endemic regions. Military personnel in Colombia frequently operate in high-malaria areas, yet the magnitude of asymptomatic infection and its associated risk factors in this population remain poorly understood. In this study, we evaluated 773 soldiers deployed in four malaria-endemic departments of Colombia using microscopy, rapid diagnostic tests, conventional PCR, and qPCR. We found that 2.59% of individuals carried asymptomatic *Plasmodium* infections, mostly caused by *P. falciparum*. Molecular methods, especially qPCR, detected infections missed by routine diagnostics. We also identified several factors associated with asymptomatic infection, including bed net use, previous malaria episodes, patrol location, and exposure duration. Our findings emphasize the importance of including molecular testing for asymptomatic malaria in military surveillance programs. Early detection and treatment of low-density infections could help reduce transmission, protect military personnel, and strengthen malaria control efforts in Colombia.

## Introduction

Malaria is a parasitic disease caused by *Plasmodium* spp. transmitted through the bite of infected female *Anopheles* mosquitoes. In humans, it is caused by five parasite species, with *P. falciparum* and *P. vivax* being the most common. The clinical presentation ranges from asymptomatic infections to severe and fatal cases [1], representing a major public health concern in which nearly half of the world’s population is at risk [2]. According to the latest World Health Organization malaria report, an estimated 249 million malaria cases occurred in 2022 across 85 countries and territories with endemic transmission. Although the Americas reported a 64% reduction in malaria cases in 2022, 73% of these originated from Venezuela, Brazil, and Colombia [3].

Colombia, due to its geographic location, topography, and climatic conditions, presents favorable environments for malaria transmission. According to the National Surveillance System, during 2024 up to epidemiological week 52, a total of 123,740 malaria cases were reported, predominantly *P. vivax* (62.6%), followed by *P. falciparum* (35.6%), mixed infections (1.8%), and no reported cases of *P. malariae*, with an overall increase in malaria incidence over the last three years [4]. This scenario underscores the need to strengthen prevention and control programs, particularly in remote rural areas and among specific population groups residing in these zones.

Malaria is one of the 12 prioritized public health events within the Colombian Military Forces. From 2019 to epidemiological week 20 of 2024, it represented the second most frequently reported vector-borne disease among active military personnel, with the highest incidence occurring in the Colombian National Army [5]. According to the Army Health Directorate, between 2015 and 2024, a total of 4,645 malaria cases occurred within this institution.

One of the main challenges in malaria control programs is the occurrence of asymptomatic and submicroscopic infections, in which individuals serve as silent reservoirs of the parasite [6]. In Colombia, the widespread distribution and high frequency of *P. vivax* contribute considerably to this phenomenon. Gametocytes of this species develop more rapidly and are transmitted more efficiently than those of *P. falciparum*, enabling individuals to infect mosquitoes before being diagnosed. Additionally, many infections occur with low parasitemia—most of them submicroscopic and asymptomatic—which remain undetected and untreated, thereby sustaining parasite reservoirs within the community [7–10]. Although some of these infections may become symptomatic days or weeks after detection, many persist [9,11].

Therefore, to achieve meaningful reductions in malaria morbidity and mortality, it is necessary to implement active surveillance strategies for asymptomatic *Plasmodium* spp. carriers so that control and prevention measures are not limited solely to symptomatic cases. In endemic regions, a high proportion of asymptomatic individuals are infected with *Plasmodium* spp., often with submicroscopic parasitemias. These individuals contribute to transmission because they may harbor gametocytes and sustain infections for longer periods than symptomatic individuals, who typically seek diagnosis and treatment [8,10,11]. To eliminate malaria, vector control and treatment of symptomatic cases must be complemented by active case detection strategies that identify asymptomatic individuals who maintain transmission [12,13].

In malaria-endemic countries, the prevalence of asymptomatic infection ranges from as high as 85% in high-transmission settings [14] to 17,6% [15] and 20% [16] in low-transmission areas such as Peru and Brazil, respectively. In areas with low endemicity, including parts of Asia and the Americas, asymptomatic infections typically present with low parasitemia, and molecular tests such as PCR are up to 50% more sensitive than microscopy for detecting these infections [7–10]. In regions where the slide positivity index is below 4%, submicroscopic infections may account for 20–50% of total transmission [11]. Beyond their role in transmission, asymptomatic infections increase the risk of chronic anemia due to the continuous destruction of infected erythrocytes.

In Colombia, few studies have investigated asymptomatic and submicroscopic infections [17–20], and the present study is the first to evaluate such infections in Colombian military personnel. Accordingly, the main objective of this work was to characterize the prevalence and risk factors associated with asymptomatic *Plasmodium* spp. infection within the Health Subsystem of the Colombian Army.

## Methods

### Ethical statement

This study was conducted in accordance with the Declaration of Helsinki and classified as minimal-risk research under Article 11 of Resolution 8430 of 1993 of the Colombian Ministry of Health. The research project was approved by the Research Ethics Committee of the Central Military Hospital, Bogotá (Minutes No. 08, May 7, 2021). Additional approvals were granted by the Local Committee of Science and Technology (DISAN; Minutes No. 178920, August 12, 2019), the Health Research Projects Committee (DIGSA; Approval No. 16231, September 18, 2019), and by authorization for project execution from the Deputy Commander of the Colombian National Army (Record OFI21–30969, April 7, 2021). All participants provided written informed consent prior to enrollment. Personal, sociodemographic, clinical, and diagnostic data were handled under strict confidentiality standards in accordance with Colombian Resolution 2378 of 2008 on Good Clinical Practices.

**Fig 1:**
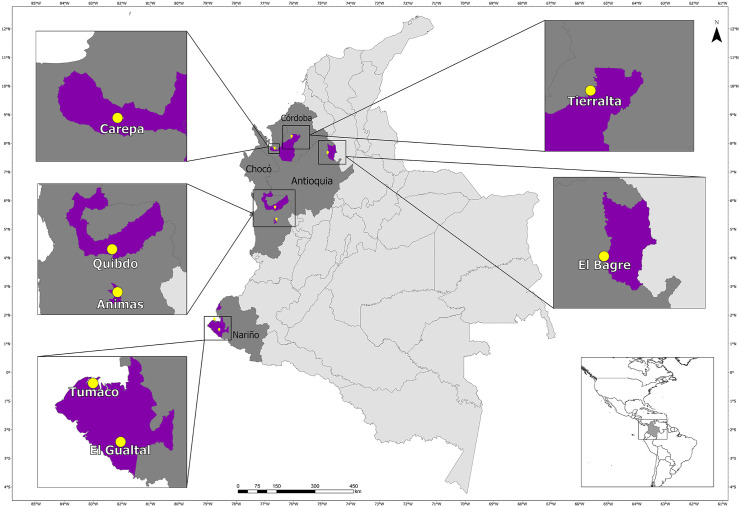
Sampling sites for asymptomatic *Plasmodium* spp. infection in Colombian military personnel. Carepa, Antioquia (n = 178), El Bagre, Antioquia (n = 52), Quibdó and Ánimas, Chocó (n = 177), Tierralta, Córdoba (n = 194), Tumaco and El Gualtal, Nariño (n = 174). This map was created in ArcGIS Pro - version 2.8 to plot the sampling coordinates with different freely accessible shapefiles of the Municipalities (source: https://ags.esri.co/arcgis/rest/services/DatosAbiertos/SERVICIOS_PUBLICOS_2005_MPIO/MapServer/0) and Departments of Colombia (source: https://ags.esri.co/arcgis/rest/services/DatosAbiertos/SERVICIOS_PUBLICOS_2005_DPTO/MapServer), as well as Global Geopolitical (source: https://services.arcgis.com/P3ePLMYs2RVChkJx/arcgis/rest/services/World_Countries_(Generalized)/FeatureServer/0) compatible with CC BY 4.0 licensing.

A stratified random sampling strategy was implemented among military personnel patrolling malaria-endemic areas in Colombia between 18 March and 1 May 2022. These personnel formed part of the troop reinforcement deployed in border regions; therefore, battalions stationed in the departments of Antioquia (El Bagre, Carepa), Chocó (Quibdó), Córdoba (Tierralta), and Nariño (Tumaco) were included, corresponding to the III and VII Divisions of the Colombian National Army (Fig 1).

An expected sample size of 806 participants from the four malaria-endemic departments was estimated using Epi Info v5.5.15 (https://www.cdc.gov/epiinfo/index.html). Calculations were based on the reference population assigned to each military unit during the sampling period, the expected prevalence for each unit or department, the corresponding margin of error (MOE), and a 95% confidence interval, following prevalence ranges reported in [17–24] (Table 1).

**Table 1:** Stratified random sampling of asymptomatic active-duty military personnel for malaria in the III and VII Divisions of the Colombian National Army.

Department	Battalion	Reference population	Expected prevalence	Absolute precision	Sample (confidence %)	Observed sample
Antioquia	Carepa	1925	5%	3%	184	177
	El Bagre	343	5%	3%	128	51
Chocó	Quibdó	935	5%	3%	167	177
Córdoba	Tierralta	761	10%	4%	169	194
Nariño	Tumaco	3713	4%	3%	158	174
**Total**					**806**	**773**

### Inclusion/exclusion criteria

Inclusion criteria were male sex, age ≥ 18 years, absence of malaria diagnosis within the previous three months, no fever or history of fever in the past 72 hours, and no antimalarial drug intake during the four weeks preceding sampling. Exclusion criteria included fever, headache, myalgia, or general malaise within the previous two weeks, as well as less than six months of active military service.

### Microscopy and RDT diagnosis of *Plasmodium* in asymptomatic patients

Capillary blood was collected onsite for microscopic and serological diagnosis using RDTs. For microscopy, thick blood smears (TBS) and peripheral blood smears were prepared according to national malaria diagnostic guidelines [25]. Parasite density for *Plasmodium* spp. as estimated on Field-stained thick smears by counting asexual and sexual stages, assuming a standard leukocyte count of 8000 cells/µL.

For RDT-based diagnosis, an ultrasensitive rapid test detecting *P. vivax* pLDH and *P. falciparum* HRP-2 (Abbott Bioline Malaria Ag Pf/Pv) was used. The performance of the ultrasensitive RDT for detecting *P. falciparum* and *P. vivax* was evaluated against qPCR molecular results.

### Molecular diagnosis by conventional PCR, nested PCR, and qPCR

DNA extraction was performed using the GeneJET Genomic DNA Purification Kit (Thermo Scientific, Vilnius, Lithuania) according to the manufacturer’s instructions. Conventional PCR (conventional PCR) for genus-level detection was carried out using primers described in [26]. Each 20 µL reaction contained 1 × Phusion U Green Multiplex PCR Master Mix, 0.3 µM of each primer, and 5 µL of extracted DNA. Genus-positive samples were subsequently subjected to species identification by nested PCR (nPCR) targeting the 18S small-subunit ribosomal RNA (ssrRNA) gene using species-specific primers for *P. falciparum*, *P. vivax*, and *P. malariae* [26].

Amplification of the 1200 bp ssrRNA fragment using primers rPLU5 and rPLU6 followed a cycling program consisting of an initial denaturation at 98°C for 30 s; 40 cycles of 98°C for 10 s, 58°C for 30 s, and 72°C for 30 s; and a final extension at 72°C for 10 min. Nested PCR reactions for species genotyping followed a similar profile, with an initial denaturation at 98°C for 30 s; 35 cycles of 98°C for 10 s, 62°C for 30 s, and 72°C for 25 s; and a final extension at 72°C for 10 min.

DNA extracted from peripheral blood was screened too for *Plasmodium* spp*.* using a genus-specific real-time PCR (qPCR) targeting a 157–165 bp fragment of the 18S rRNA gene, with an endogenous internal control (ERV-3; 135 bp), followed by a species-specific multiplex qPCR for *P. falciparum*, *P. vivax*, and *P. malariae* as previously described [27,28]. All primers and probes used in the conventional PCR and qPCR assays are listed in S1 Table. Primers and probes used for conventional PCR and qPCR detection of *Plasmodium* spp. The screening qPCR (18S + ERV-3) was performed in a final volume of 20 µL containing 5 µL of DNA, 0.3 µM of each primer, 0.2 µM of each probe, and 1 × Luminaris Color Probe qPCR Master Mix (Thermo Scientific), with a UDG pre-treatment at 50°C for 2 min, initial denaturation at 95°C for 10 min, followed by 45 cycles of 95°C for 15 s and 60°C for 1 min. Samples with no amplification signal before cycle 40 (Ct ≥ 40) were considered negative. Species identification was carried out using the same thermal profile and primer pair (Plasmo 1/Plasmo 2), combined with three species-specific probes for *P. falciparum*, *P. vivax*, and *P. malariae* (S1 Table).

Genomic DNA from *Plasmodium falciparum* was obtained from a strain donated by the Programa de Estudio y Control de Enfermedades Tropicales (PECET) at Universidad de Antioquia and was used along with a DNA extract from a patient with a confirmed diagnosis of *Plasmodium vivax* infection, as positive controls in all molecular assays. Additionally, conventional PCR, nPCR and qPCR included a Not Template Control (NTC) to assess the quality of the PCR Master Mix reagents and a Negative Control to assess the purity of the extraction reagents. All samples were validated in duplicate through intra and inter-assay in order to evaluate reproducibility

### Sanger sequencing

PCR products were resolved on 2% agarose gels in 1 × TAE buffer, stained with GelRed, and electrophoresed at 100 V for 50 min. Bands were visualized on a Gel Doc XR+ system (Bio-Rad, CA, USA), and presence/absence of amplicons was used for molecular diagnosis. Genus-level PCR products (~1200 bp) were selected for Sanger sequencing. Amplicons of 1050 bp from the ssrRNA gene were purified using ExoSAP-IT (Applied Biosystems, Thermo Fisher Scientific) and sequenced with BigDye Terminator v3.1 chemistry on an ABI 3730xl DNA Analyzer (Macrogen, South Korea).

### Bioinformatic analyses

Sequences were edited using Geneious Prime [29], and evaluated using BLASTn (http://blast.ncbi.nlm.nih.gov) to confirm infecting *Plasmodium* species.

### Gold standard for sensitivity in asymptomatic patients

qPCR was used as the gold standard for detecting parasitemia below 0.02 parasites/µL, previously established for asymptomatic malaria infection via conventional PCR [30].

### Biostatistical analyses

All descriptive, univariate, and multivariate analyses were performed using IBM SPSS Statistics v26. Normality was assessed using the Kolmogorov–Smirnov test (n > 50), and nonparametric tests were applied when distributional assumptions were not met. Categorical variables were analyzed using Pearson’s Chi-square test, and quantitative variables using the Mann–Whitney U test. All tests were two-tailed, with statistical significance defined as p < 0.05 (*) and high statistical significance as p < 0.01 (**). Following epidemiological criteria and the recommendations of Hosmer and Lemeshow for variable selection in multivariate modeling, p-values < 0.21 were also considered based on parsimony and biological plausibility [31–33].

Principal Component Analysis (PCA) was performed on the variables age, number of lifetime malaria episodes, number of episodes in the last two years, days of patrol, number of patrol sites, frequency of bed net use, and frequency of repellent use. The analysis was conducted using correlation matrices with varimax orthogonal rotation and anti-image correlation diagnostics. Sampling adequacy and suitability for PCA were evaluated using the Kaiser–Meyer–Olkin (KMO) measure and Bartlett’s test of sphericity. Variables were considered to contribute meaningfully to a component when factor loadings were greater than 0.4 or less than –0.4.

## Results

### Sampling

Once fieldwork was completed, a total of 773 samples meeting the eligibility criteria were collected. The expected sample size was achieved at 95.91%, primarily due to the limited accessibility of some military units, operational commitments of personnel in active patrol areas, and public order constraints in Carepa and El Bagre (Antioquia), where sampling reached 96.20% and 39.84%, respectively. Owing to these logistical challenges, sampling thresholds were exceeded in Quibdó, Tierralta, and Tumaco, with observed sampling reaching 105.99%, 114.79%, and 110.13%, respectively. Raw data are provided in the Supporting Information (S2 Table)

### Microscopy and RDT diagnosis of *Plasmodium* in asymptomatic patients

Microscopic diagnosis using thick blood smears and peripheral blood smears detected *Plasmodium* positivity in 0.26% (2/773) of samples, corresponding to 5520 asexual forms/µL of *P. vivax* in patient 526 from Carepa (Antioquia), and a single *P. falciparum* gametocyte in patient 713 from El Bagre (Antioquia). In contrast, all individuals tested negative by rapid diagnostic tests, yielding a prevalence of 0% (0/773) using this method.

### Molecular diagnosis by conventional PCR, nested PCR, and qPCR

For molecular diagnosis, conventional PCR detected *Plasmodium* spp*.* in 2.59% (20/773) of sampled individuals. Nested PCR enabled species-level genotyping in 100% (20/20) of conventional PCR-positive samples. Genus-level qPCR detection showed complete agreement with conventional PCR (2.59%), and species identification by qPCR was successful in 95% (19/20) of qPCR-positive samples.

### Sanger sequencing

Sanger sequencing successfully identified the infecting species in 60% (12/20) of positive cases using PCR products of 986–1031 bp, whereas the remaining 40% (8/20) were successfully sequenced using shorter PCR products of 121–206 bp. All genetic sequences generated in this study have been deposited in GenBank under accession numbers PQ559777–PQ559785.

### Diagnostic sensitivity

Considering qPCR as the gold standard, the sensitivity of the diagnostic methods was 10% for thick blood smear and peripheral smear, 0% for RDTs, and 100% for conventional PCR (Table 2).

**Table 2:** Positive patients with asymptomatic malaria infection according to microscopy, RDT, and molecular diagnostic methods.

Patient	RDT	Microscopy	cPCR *18S*	Sanger Sequencing (986 bp - 1031 bp)	Species nested PCR		Genus *Plasmodium* spp.		Species genotypification		
						Sanger Sequencing (121 bp - 206 bp)	*18S* FAM (*Ct*)	*ERV-3* Cy5 (*Ct*)	*P. falciparum* FAM (*Ct*)	*P. vivax* HEX (*Ct*)	*P. malariae* RED 610 (*Ct*)
519 C	Neg	Neg	+	*–*	*P. vivax*	*P. vivax*	39.27	22.12	–	38.40	N/A
526 C	Neg	*P. vivax* 5220 asexual forms/µL blood.	+	*P. vivax* PQ559781	*P. falciparum - P. vivax*	N/A	33.56	21.10	35.58	–	N/A
647 C	Neg	Neg	+	*P. vivax* PQ559785	*P. vivax*	N/A	31.85	19.08	–	31.57	N/A
651 C	Neg	Neg	+	*P. falciparum* PQ559778	*P. falciparum*	N/A	33.73	21.22	31.81	–	N/A
664 C	Neg	Neg	+	*–*	*P. falciparum -P. vivax*	*P. vivax*	38.34	24.71	37.34	–	N/A
676 C	Neg	Neg	+	*P. falciparum* PQ559777	*P. falciparum*	N/A	38.20	25.55	34.31	–	N/A
517 Ch	Neg	Neg	+	*–*	*P. falciparum*	*P. falciparum*	37.37	23.20	35.89	–	N/A
605 Ch	Neg	Neg	+	*P. falciparum* PQ559780	*P. falciparum*	N/A	34.36	21.56	32.55	–	N/A
644 Ch	Neg	Neg	+	*. vivax* PQ559784	*P. vivax*	N/A	33.58	21.63	–	31.36	N/A
713 B	Neg	*P. falciparum* 1 gametocyte	+	*–*	*P. falciparum*	*P. falciparum*	35.29	21.97	36.08	–	N/A
719 B	Neg	Neg	+	*P. falciparum* PQ559779	*P. falciparum*	N/A	33.68	21.48	32.32		N/A
724 B	Neg	Neg	+	*P. vivax* PQ559782	*P. vivax*	N/A	32.57	20.39	–	‘	N/A
735 B	Neg	Neg	+	*P. vivax* PQ559783	*P. vivax*	N/A	34.31	22.24	–	33.78	N/A
744 B	Neg	Neg	+	*–*	*P. falciparum*	*P. falciparum*	35.17	20.73	35.58	–	N/A
514 TA	Neg	Neg	+	*P. falciparum* PQ559778	*P. falciparum*	N/A	36.33	24.17	32.16	–	N/A
674 TA	Neg	Neg	+	–	*P. falciparum -P. vivax*	*P. vivax*	37.63	23.29	37.39	–	N/A
687 TA	Neg	Neg	+	*P. falciparum* PQ559777	*P. falciparum*	N/A	36.22	23.76	33.27	–	N/A
514 T	Neg	Neg	+	*P. falciparum* PQ559778	*P. falciparum*	N/A	36.40	23.83	31.95	–	N/A
582 T	Neg	Neg	+	*–*	*P. falciparum*	*P. falciparum*	37.07	22.16	36.88	–	N/A
617 T	Neg	Neg	+	*–*	*P. falciparum*	*P. falciparum*	35.94	22.94	32.91	–	N/A

Rapid Diagnostic Test (RDT); Conventional PCR (cPCR); base pair (bp); Cycle threshold (*Ct*); Carepa (C); Chocó (Ch); Bagre (B); Tierralta (TA); Tumaco (T); Not Applicable (N/A); Negative (Neg).

### Prevalence of asymptomatic *Plasmodium* spp. infection

Once the diagnostic methods were compared, this study documented a prevalence of asymptomatic *Plasmodium* spp. infection of 2.59% among military personnel stationed in the four border departments with the highest malaria endemicity (Antioquia, Chocó, Córdoba, and Nariño) during 2022, as determined by conventional PCR and qPCR. Likewise, the prevalence of asymptomatic infection based on these diagnostic methods was 9.80% (6/51) in El Bagre–Antioquia, 3.39% (6/177) in Carepa–Antioquia, 1.72% (3/174) in Tumaco–Nariño, 1.69% (3/177) in Quibdó–Chocó, and 1.55% (3/194) in Tierralta–Córdoba.

Among asymptomatic infections, *P. falciparum* accounted for 60% (12/20), *P. vivax* for 25% (5/20), and mixed infections for 15% (3/20) (Table 2). By sampling site, species-specific prevalence was as follows: in El Bagre–Antioquia, *P. falciparum* constituted 60% (3/5) and *P. vivax* 40% (2/5); in Carepa–Antioquia, *P. falciparum* accounted for 33.34% (2/6), *P. vivax* for 33.33% (2/6), and mixed infections for 33.33% (2/6); in Tumaco–Nariño, *P. falciparum* was detected in 100% (3/3); in Quibdó–Chocó, *P. falciparum* represented 66.67% (2/3) and *P. vivax* 33.33% (1/3); and in Tierralta–Córdoba, *P. falciparum* accounted for 66.67% (2/3) and mixed infections for 33.33% (1/3).

### Risk factors associated with asymptomatic malaria infection

For the quantitative variables—including age (K–S = 0.225; p = 0.000), number of lifetime malaria episodes (K–S = 0.442; p = 0.000), number of malaria episodes in the last two years (K–S = 0.500; p = 0.000), parasite count by thick smear (K–S = 0.512; p = 0.000), days of patrol (K–S = 0.182; p = 0.000), number of patrol sites (K–S = 0.446; p = 0.000), frequency of bed net use (K–S = 0.276; p = 0.000), and frequency of repellent use (K–S = 0.303; p = 0.000)—the Kolmogorov–Smirnov test (n = 773) indicated p < 0.05 for all variables, demonstrating that the data did not follow a normal distribution (Table 3). Accordingly, the nonparametric Mann–Whitney U test was applied, and statistically significant differences (p < 0.05) between healthy individuals and those with asymptomatic malaria infection were found only for the variable “frequency of bed net use” (U = 5616.0; p = 0.040).

**Table 3:** Quantitative demographic and clinical-epidemiological data in healthy and asymptomatic patients.

	Age		Number of lifetime malaria episodes		Number of malaria episodes in the last two years	
	Asymptomatic n = 20	Healthy n = 753	Asymptomatic n = 20	Healthy n = 753	Asymptomatic n = 20	Healthy n = 753
U M-W	6917.5 (*p* = 0.531)		**6558.5 (*p* = 0.159) †**		**6858.0 (*p* = 0.204) †**	
**Mean**	23.35	23.43	0,40	0,40	0,20	0,16
**Median**	21.00	21.00	0,00	0,00	0,00	0,00
**Variance**	31.82	35.59	0.36	1.18	0.17	0.336
**Minimum**	19	18	0	0	0	0
**Maximum**	39	41	2	10	1	7
	**Days of patrol**			**Number of patrol sites**		
	Asymptomatic n = 19	Healthy n = 698		Asymptomatic n = 19	Healthy n = 697	
U M-W	5548.5 (*p* = 0.224)			6061.0 (*p* = 0.419)		
**Mean**	113.84	110.53		1.37	1.30	
**Median**	100.00	95.00		1.00	1.00	
**Variance**	3585.90	9078.30		0.25	0.25	
**Minimum**	17	0		1	1	
**Maximum**	217	1105		2	3	
	**Frequency of bed net use**			**Frequency of repellent use**		
	Asymptomatic n = 20	Healthy n = 753		Asymptomatic n = 20	Healthy n = 753	
U M-W	5616.0 (*p* = 0.040) *			7455.5 (*p* = 0.935)		
**Mean**	2.55	1.72		1.25	1.30	
**Median**	3.00	1.00		0.50	0.00	
**Variance**	2.47	3.01		2.30	2.40	
**Minimum**	0	0		0	0	
**Maximum**	4	4		4	4	

* Significance according to statistical criterion p < 0.05.

† Significance according to epidemiological criterion, principle of parsimony, and biological plausibility p < 0.21.

The Mann–Whitney U test (U-M-W).

**Fig 2:**
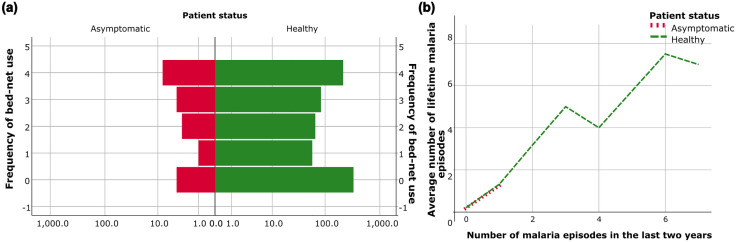
Preventive practices and malaria history among healthy and asymptomatic military personnel. **(a)** Frequency of bed net use among healthy (n = 753) and asymptomatic participants (n = 20), displayed on a logarithmic scale (log₁₀). Categories: Never = 0, Rarely = 1, Occasionally = 2, Frequently = 3, and Very frequently = 4. **(b)** Association between lifetime malaria episodes and malaria episodes reported in the last two years among healthy and asymptomatic participants. Mean lifetime malaria episodes are plotted against the number of episodes in the past two years.

Healthy individuals most frequently reported never using a bed net (n = 331; frequency category f = 0), followed by very frequent use (f = 4; n = 214). In contrast, asymptomatic patients primarily reported very frequent use (n = 8; f = 4), followed by never (n = 4; f = 0) and frequent use (n = 4; f = 3). Consequently, the mean bed net use was 1.72 among healthy individuals compared with 2.55 in asymptomatic patients, suggesting greater engagement in preventive measures among the latter (see Fig 2a).

However, following theoretical recommendations by statistical epidemiologists—who advise interpreting results based not only on statistical significance but also on epidemiological relevance—criteria such as the Hosmer & Lemeshow significance threshold (p < 0.10), the principle of parsimony (p < 0.21), and biological plausibility based on expert judgment indicate that the variables “number of lifetime malaria episodes” (U = 6558.5; p = 0.159) and “number of malaria episodes in the last two years” (U = 6858.0; p = 0.204) may also represent risk factors associated with asymptomatic malaria infection. Accordingly, healthy individuals reported a higher number of lifetime malaria episodes and more episodes in the last two years compared with asymptomatic patients (see Fig 2b).

With respect to qualitative variables—including ethnic background, military rank, department of birth, department of origin, department of patrol, patrol unit, displacement in the last 15 days, and contact with symptomatic individuals—the nonparametric Pearson’s Chi-square test was applied. This analysis revealed highly significant differences between healthy and asymptomatic individuals for the variable “department of patrol” (Chi² = 40.23; df = 14; p = 0.000). Additionally, based on epidemiological criteria, the principle of parsimony, and biological plausibility according to expert judgment, the variable “department of origin” (Chi² = 7.80; df = 4; p = 0.099) was also considered a potential risk factor associated with asymptomatic malaria infection (Table 4).

**Table 4:** Qualitative demographic and clinical-epidemiological data in healthy and asymptomatic patients.

Variable	Asymptomatic		Healthy		Pearson Chi^2^
Ethnic background	Frequency	%	Frequency	%	
*Indigenous*	1	5.0	15	2.0	1.189 (*p* = 0.552)
*Mestizo*	16	80.0	653	86.7	
*NMA*	3	15.0	85	11.3	
***Total***	**n = 20**	**100.0**	**n = 753**	**100.0**	
**Military rank**	**Frequency**	**%**	**Frequency**	**%**	
*SLR*	12	60.0	445	59.1	7.086 (*p* = 0.717)
*SLP*	5	25.0	264	35.1	
*C3*	1	5.0	12	1.6	
*CS*	1	5.0	9	1.2	
*CP*	0	0.0	8	1.1	
*SS*	1	5.0	8	1.1	
*SV*	0	0.0	1	0.1	
*ST*	0	0.0	3	0.4	
*TE*	0	0.0	1	0.1	
*MY*	0	0.0	1	0.1	
*TC*	0	0.0	1	0.1	
**Total**	**n = 20**	**100.0**	**n = 753**	**100.0**	
**Department of birth**	**Frequency**	**%**	**Frequency**	**%**	
*Amazonas*	0	0.0	1	0.1	20.648 (*p* = 0.939)
*Antioquia*	4	20.0	160	21.2	
*Atlántico*	0	0.0	35	4.6	
*Bogotá*	2	10.0	13	1.7	
*Bolívar*	2	10.0	38	5.0	
*Boyacá*	0	0.0	12	1.6	
*Caldas*	0	0.0	5	0.7	
*Caquetá*	0	0.0	11	1.5	
*Casanare*	0	0.0	3	0.4	
*Cauca*	1	5.0	26	3.5	
*Cesar*	1	5.0	12	1.6	
*Choco*	2	10.0	58	7.7	
*Cundinamarca*	0	0.0	12	1.6	
*Córdoba*	5	25.0	173	23.0	
*Guaviare*	0	0.0	2	0.3	
*Huila*	0	0.0	12	1.6	
*La Guajira*	0	0.0	4	0.5	
*Magdalena*	0	0.0	24	3.2	
*Meta*	0	0.0	4	0.5	
*Monteria*	0	0.0	1	0.1	
*Nariño*	0	0.0	16	2.1	
*Norte de Santander*	0	0.0	7	0.9	
*Putumayo*	0	0.0	11	1.5	
*Quibdó*	0	0.0	1	0.1	
*Quindío*	0	0.0	1	0.1	
*Risaralda*	1	5.0	5	0.7	
*Santander*	0	0.0	15	2.0	
*Sucre*	2	10.0	46	6.1	
*Tolima*	0	0.0	22	2.9	
*Valle Del Cauca*	0	0.0	23	3.0	
***Total***	**n = 20**	**100.0**	**n = 753**	**100.0**	
**Department of origin**	**Frequency**	**%**	**Frequency**	**%**	
*Antioquia*	11	55.0	207	27.5	**7.799** **(*p* = 0.099) †**
*Chocó*	2	10.0	157	20.8	
*Cundinamarca*	1	5.0	27	3.6	
*Córdoba*	3	15.0	191	25.4	
*Nariño*	3	15.0	171	22.7	
***Total***	**n = 20**	**100.0**	**n = 753**	**100.0**	
**Department of patrol**	**Frequency**	**%**	**Frequency**	**%**	
*Antioquia*	11	55.0	339	45.0	**40.227** (*p* = 0.000) **
*Chocó*	5	25.0	170	22.6	
*Chocó-Antioquia*	0	0.0	9	1.2	
*Chocó-Guajira*	0	0.0	1	0.1	
*Cundinamarca*	0	0.0	2	0.3	
*Cundinamarca-Chocó*	0	0.0	1	0.1	
*Córdoba-Antioquia*	0	0.0	2	0.3	
*Córdoba-Chocó*	1	5.0	0	0.0	
*Córdoba-Chocó-Antioquia*	0	0.0	1	0.1	
*Córdoba*	0	0.0	1	0.1	
*Nariño*	2	10.0	163	21.6	
*Risaralda-Nariño*	0	0.0	1	0.1	
*Valle del Cauca*	0	0.0	3	0.4	
*Valle del Cauca -Chocó*	0	0.0	7	0.9	
*No data*	1	5.0	53	7.0	
***Total***	**n = 20**	**100.0**	**n = 753**	**100.0**	
**Patrol unit**	**Frequency**	**%**	**Frequency**	**%**	
*ALTAMONTA3*	0	0.0	1	0.1	7.438 (*p* = 1.000)
*BACN4*	0	0.0	28	3.7	
*BADRA6*	1	5.0	76	10.1	
*BAEEV5*	8	40.0	228	30.3	
*BAFER*	1	5.0	22	2.9	
*BASENAVAL*	0	0.0	2	0.3	
*BASENAVAL-BAFER*	0	0.0	2	0.3	
*BATOT14*	1	5.0	23	3.1	
*BATOT15*	0	0.0	21	2.8	
*BATOT16*	0	0.0	16	2.1	
*BATOT25*	0	0.0	2	0.3	
*BATOT26*	0	0.0	4	0.5	
*BATRIFLES*	0	0.0	1	0.1	
*BIBEM17*	1	5.0	50	6.6	
*BIJUL15*	1	5.0	35	4.6	
*BIPLI12*	1	5.0	49	6.5	
*BITER15*	0	0.0	27	3.6	
*BIVEL47*	1	5.0	18	2.4	
*BIVOL46*	3	15.0	60	8.0	
*CDELDARIEN*	0	0.0	1	0.1	
*CORRUGADOS*	0	0.0	2	0.3	
*DETONADOR4*	0	0.0	4	0.5	
*EMSUB*	0	0.0	2	0.3	
*GRULI9*	0	0.0	24	3.2	
*PTOVALDIVIA*	0	0.0	2	0.3	
*No data*	2	10.0	53	7.0	
***Total***	**n = 20**	**100.0**	**n = 753**	**100.0**	
**Displacement in the last 15 days**	**Frequency**	**%**	**Frequency**	**%**	
*No*	**17**	**85.0**	564	74.9	1.074 (*p* = 0.584)
*Yes*	**3**	**15.0**	188	25.0	
*No data*	0	0.0	1	0.1	
***Total***	**n = 20**	**100.0**	**n = 753**	**100.0**	
**Contact with symptomatic individuals**	**Frequency**	**%**	**Frequency**	**%**	
*No*	18	90.0	659	87.5	1.017 (*p* = 0.601)
*No data*	0	0.0	34	4.5	
*Yes*	2	10.0	60	8.0	
***Total***	**n = 20**	**100.0**	**n = 753**	**100.0**	

** Significance according to statistical criterion p < 0.01.

† Significance according to epidemiological criterion, principle of parsimony, and biological plausibility p < 0.21.

**Fig 3:**
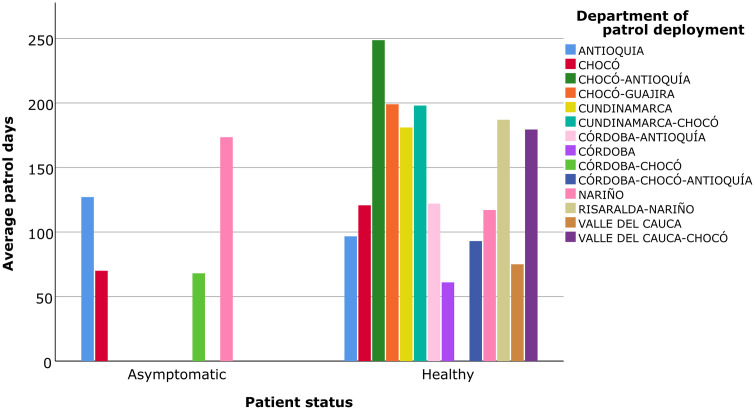
Patrol days by deployment department in healthy and asymptomatic participants. Average number of patrol days reported by healthy and asymptomatic participants, stratified by the department of military patrol deployment.

Considering that military personnel patrolled in one or more departments, the categories within the variable “department of patrol” showed significant differences between healthy and asymptomatic individuals. Notably, departments with low malaria endemicity—such as Cundinamarca, Risaralda, and Valle del Cauca—appeared exclusively among healthy individuals, whereas patrolling in Córdoba–Chocó was reported only among asymptomatic individuals (Fig 3). Although the number of patrol days did not differ significantly between groups, a greater number of patrol days—and therefore greater exposure—in Antioquia and Nariño was associated with a higher prevalence of asymptomatic malaria infection. This was supported by the finding that asymptomatic individuals (X̅ = 113.84; n = 19) spent, on average, more days patrolling than healthy individuals (X̅ = 110.53; n = 698).

Although healthy individuals reported higher absolute numbers of lifetime malaria episodes and malaria episodes in the last two years—representing 19.79% (n = 149) and 10.49% (n = 79), respectively—35% (n = 7) of asymptomatic individuals reported at least one previous malaria episode, and 20% (n = 4) reported at least one episode in the last two years. Proportionally, a higher percentage of asymptomatic individuals had experienced malaria at least once, both in their lifetime and in recent years, compared with healthy individuals. Mean values reflected this pattern: both groups had a mean of X̅ = 0.40 lifetime malaria episodes, but the mean number of episodes in the last two years was higher among asymptomatic individuals (X̅ = 0.20) than among healthy individuals (X̅ = 0.16).

**Fig 4:**
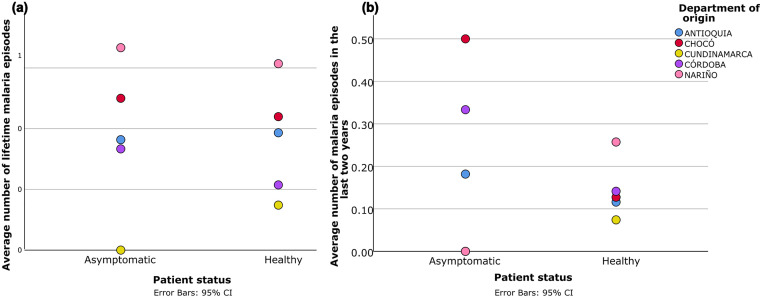
Malaria episodes by department of origin among healthy and asymptomatic military personnel. **(a)** Mean number of lifetime malaria episodes stratified by department of origin. **(b)** Mean number of malaria episodes in the past two years stratified by department of origin. Error bars represent 95% confidence intervals.

When the average number of lifetime malaria episodes was evaluated by department of origin, asymptomatic individuals had more lifetime episodes in Chocó, Córdoba, and Nariño (Fig 4a). Likewise, when the average number of malaria episodes in the last two years was assessed by department of origin, Antioquia, Chocó, and Córdoba showed a greater number of episodes among asymptomatic individuals (Fig 4b).

For the PCA, seven quantitative variables were included (age, number of lifetime malaria episodes, number of episodes in the last two years, days of patrol, number of patrol sites, frequency of bed net use, and frequency of repellent use). The first three principal components explained 73.69% of the total variance. Sampling adequacy was confirmed with the Kaiser–Meyer–Olkin test (KMO = 0.658), for which values > 0.6 are recommended (Méndez et al., 2020), and Bartlett’s test of sphericity (X² = 1311.68; df = 21; p = 0.000), indicating high correlation among variables.

**Fig 5:**
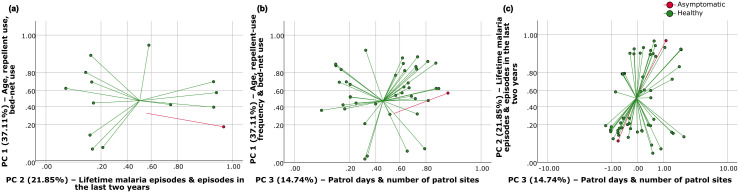
Principal component analysis of risk factors associated with asymptomatic malaria infection. Principal component analysis (PCA) of risk factors associated with asymptomatic malaria infection. PC1 explains 37.11% of the variance, PC2 explains 21.85%, and PC3 explains 14.74%. **(A)** PCA of PC1 and PC2 (58.96% cumulative variance), showing clear separation between the centroids of healthy and asymptomatic participants. **(B)** PCA of PC1 and PC3 (51.85% cumulative variance), also demonstrating differences between the centroids of both groups. **(C)** PCA of PC2 and PC3 (36.59% cumulative variance), showing no separation between the group centroids.

According to the rotated component matrix (Varimax with Kaiser normalization), the variables “frequency of repellent use” (0.869), “frequency of bed net use” (0.804), and “age” (0.751) showed loadings > 0.5 in PC1. In PC2, the variables “number of lifetime malaria episodes” (0.901) and “number of malaria episodes in the last two years” (0.909) also showed loadings > 0.5, while in PC3, loadings > 0.5 were observed for “days of patrol” (0.914) and “number of patrol sites” (0.669) (Fig 5). Accordingly, the variables grouped in PC1 (preventive measures and age) and PC2 (malaria episode history) explained substantial differences between healthy and asymptomatic individuals (Fig 5A). The variables in PC3 (days of patrol and number of patrol sites), together with those in PC1, also explained variation between the groups (Fig 5B). Finally, the scatter plot of PC2 versus PC3 showed no clear separation between healthy and asymptomatic individuals (Fig 5C).

## Discussion

This study in the Colombian Army highlights that asymptomatic malaria infection plays a significant role in sustaining malaria transmission, with a prevalence of 2.59% according to qPCR and conventional PCR, 0.26% by microscopy, and 0% by RDT in the departments of Antioquia, Chocó, Córdoba, and Nariño. The findings suggest that interventions such as epidemiological containment, timely diagnosis, and treatment of human reservoirs could reduce future morbidity and mortality from this vector-borne disease (VBD) in military personnel. Additionally, several risk factors associated with asymptomatic malaria infection were identified: bed net use frequency, number of lifetime malaria episodes, number of episodes within the last two years, department of origin, and department of patrol deployment (from univariate analyses), as well as age, days of patrol, and number of patrol sites (from multivariate analyses).

In military personnel from Papua New Guinea (n = 233), Lao (n = 313), Iran (n = 300), and Vietnam (n = 1223), prevalence by conventional PCR and nPCR was 24.06%, 10.87%, 1.30%, and 1.00%, respectively [34–37]. These findings illustrate the marked heterogeneity of asymptomatic malaria prevalence across military populations. This study is the first to evaluate the prevalence of asymptomatic malaria infection in a military population using qPCR. Differences in transmission intensity, prior exposure, and acquired immunity likely explain part of this variability.

Because asymptomatic infections are dynamic and may progress to symptomatic disease, longitudinal studies with standardized criteria are needed to better characterize transmission reservoirs. Compared with previous studies in Colombia, the prevalence in active military personnel was lower than that reported in civilian populations: 10% and 9.7% (n = 1169) by qPCR in Córdoba, Nariño, and Valle del Cauca in 2011 and 2015 [19,21], and 5.3% (n = 787) by PCR in pregnant women in 2016 in Antioquia, Chocó, and Nariño [22]. These comparisons suggest that asymptomatic malaria may occur at lower frequency in this military cohort than in previously studied civilian groups.

At the municipal scale, the prevalences detected in Tierralta–Córdoba and Tumaco–Nariño were lower than those previously reported for these localities, which ranged from 13.5% to 14.6% [17,19,21] and from 3.4% to 12% [19–21], respectively. In contrast, the prevalence observed in Quibdó/Ánimas–Chocó was consistent with earlier findings, which varied from 0% by microscopy (n = 223) to 2.64% by PCR (n = 227) in civilian populations and pregnant women [18,22]. Finally, in El Bagre/Carepa–Antioquia, the prevalences recorded here exceeded previously published PCR-based reference values of 0.7% (Apartadó/El Bagre/Turbo; n = 285), 0.97% (Turbo/Necoclí/San Pedro/Mulata; n = 399), and 1.64% (El Bagre/Turbo; n = 713) for pregnant women, civilians, and Indigenous populations, respectively [22–24]. Overall, municipal-level differences underscore the need for expanded and standardized sampling to accurately estimate local transmission intensity.

To date, all studies of asymptomatic malaria infection conducted in Colombia have focused on civilian populations. Consequently, direct comparison with the present study has limited biological relevance, as the military personnel evaluated here were exclusively men within a narrow age range (18–39 years; X̅ = 23.35), with no representation of children, older adults, or pregnant women, and with distinct sociodemographic characteristics.

According to the INS, *Plasmodium vivax* remains the predominant parasite species contributing to malaria transmission in Colombia, followed by *Plasmodium falciparum* and mixed infections. In this study, however, *P. falciparum* was the most prevalent species in asymptomatic infections across Antioquia (Carepa and El Bagre), Córdoba (Tierralta), Chocó (Quibdó), and Nariño (Tumaco). This pattern is consistent with previous studies conducted in Colombia, where *P. falciparum* accounted for more than half of asymptomatic infections in some endemic settings [24,38]. However, other studies conducted in Antioquia, Córdoba, and Nariño have identified *P. vivax* as the predominant species, underscoring regional and temporal variability in species distribution [19,20,22–24].

According to epidemiological studies of asymptomatic malaria infection in Colombia, the Pacific region (departments of Chocó, Cauca, Nariño, and Valle del Cauca) is characterized by a higher prevalence of *P. falciparum*, whereas *P. vivax* predominates in the Andean (Antioquia) and Caribbean (Córdoba) regions [39,40]. Limited sampling in some municipalities may also have influenced the observed species distribution.

In Tierralta, *P. falciparum* was identified as the most prevalent species, despite earlier studies reporting *P. vivax* as the predominant parasite in the department of Córdoba [19,20,38]. This discrepancy may reflect temporal shifts in vector composition and transmission patterns [40]. In contrast, the findings for Tumaco and Quibdó are consistent with previous reports describing *P. falciparum* as the dominant species in asymptomatic malaria infections in the departments of Chocó and Nariño [19,20,22]. Because military personnel operate in forested environments, their exposure to sylvatic and zoophilic vectors may differ from that of civilian populations, potentially influencing species-specific transmission patterns [34,37,41,42].

The diagnostic sensitivity results for microscopy and RDTs presented here align with earlier studies documenting poor performance of these methods in patients with low parasitemia and in those with subpatent or submicroscopic infections, including individuals with asymptomatic malaria [17,20,43,44]. This limitation hampers malaria elimination efforts in highly endemic regions by allowing transmission foci to persist due to reservoirs that remain undetected by standard diagnostics but can be identified through more sensitive molecular techniques such as conventional PCR, nPCR, and qPCR [19,21].

The absence of RDT-positive asymptomatic cases in this cohort may reflect limited sensitivity at low parasite densities and the relatively small sample size, as previously observed in other military populations [37]. Low parasite densities likely contributed to false-negative RDT results, consistent with prior reports [44]. Differences across studies may also relate to variability in RDT brand performance, highlighting the need for comparative evaluations of diagnostic sensitivity in asymptomatic infections [35]. Despite these limitations, RDTs remain operationally valuable tools in field settings.

The false-negative results obtained by microscopy (2.33%; 18/ 773) further illustrate the limitations of this diagnostic approach, which typically detects only parasitemias exceeding 50–100 parasites/µL under field conditions [45]. This raises important concerns regarding the use of thick blood smear as the gold standard for detecting asymptomatic *Plasmodium* spp. infection [30], particularly because qPCR and conventional PCR identified ten times more cases than microscopy in this study. Only two asymptomatic individuals were microscopy-positive, illustrating the limited capacity of conventional methods to detect low-density infections. These cases underscore the complexity of distinguishing persistent, recrudescent, or new infections in asymptomatic individuals and reinforce the importance of standardized inclusion criteria in future studies [9].

Regarding risk factors for asymptomatic malaria infection in Colombian military personnel, the association with the number of lifetime malaria episodes and episodes within the last two years is consistent with previous studies. In high-transmission settings, repeated exposure promotes partial immunity, increasing the likelihood of asymptomatic infections [9]. This supports the idea that adults from highly endemic areas who experienced one or more malaria episodes in childhood or adolescence are more likely to develop natural immunity and subsequently present with asymptomatic infections [20].

In terms of preventive measures, this study identified bed net use frequency as a risk factor, consistent with findings from [42,46]. Asymptomatic individuals reported higher bed net use, suggesting that transmission may occur outside typical sleeping hours, particularly during dusk patrol activities when vector biting peaks [47]. Previous studies in military settings indicate that pharmacological prophylaxis may offer greater protection than personal protective measures alone [35,48].

Department of origin also emerged as a significant risk factor. In Antioquia, asymptomatic cases were found at double the proportion observed among healthy individuals, whereas the opposite trend was noted in Chocó, Córdoba, and Nariño. These patterns suggest heterogeneous transmission intensity across departments. This interpretation aligns with the prevalence patterns observed here, in which Carepa and El Bagre exhibited higher rates of asymptomatic malaria than Quibdó, Tierralta, and Tumaco. Interestingly, national surveillance data reported higher symptomatic case numbers in Chocó, Nariño, and Córdoba than in Antioquia in 2022 [49], underscoring differences between civilian and military transmission patterns. Because department of origin likely reflects the probable site of infection for military personnel, these findings correspond with the “district of residence” risk factor identified in a study of asymptomatic malaria conducted in Vietnam [37]. Although some participants reported Cundinamarca as their department of origin, this non-endemic region likely reflects prior deployment history rather than true infection risk (Fig 4).

To our knowledge, no previous studies have formally evaluated patrol-related variables as risk factors for asymptomatic malaria in military populations [34–37,42,50]. In contrast, our findings indicate that the department in which patrols were conducted was a significant risk factor. Asymptomatic individuals were more likely to patrol in high-transmission departments, reinforcing the role of operational exposure in infection risk. Similarly, the variables “days of patrol” and “number of patrol sites,” which emerged as risk factors in the multivariate analyses, have not been previously evaluated in military populations. These findings are consistent with civilian studies showing that longer residence in endemic areas increases the likelihood of asymptomatic infection [20,23], Consistent with this, our results show that soldiers who spend more days patrolling and/or patrol a greater number of locations are more likely to acquire asymptomatic malaria than those who patrol less frequently or in fewer sites (Table 3).

The final variable identified as a significant risk factor in the multivariate analysis was patient age, consistent with observations from military studies in other countries. Age-related patterns have also been described in other military populations [37,42,50]. Age plays a similarly important role in civilian populations. In endemic settings, immunity develops with cumulative exposure, modifying both parasitemia and symptomatology across age groups [42,51].

Although age has not consistently emerged as a risk factor in previous Colombian studies [19,20,24], our findings suggest that even within a relatively homogeneous adult cohort, age-related differences may influence asymptomatic infection risk. Within this relatively narrow age range, asymptomatic individuals were slightly younger on average than healthy participants (Table 3), consistent with established patterns in which high-parasitemia infections decline with age and submicroscopic infections predominate as immunity matures [9].

Some risk factors previously described in civilian populations were not significant in this military cohort, likely reflecting its specific sociodemographic profile. In civilian settings, sex has been reported as a risk factor for asymptomatic malaria infection because rural outdoor activities typically performed by men increase their exposure to mosquito bites compared with women [20]. Sex could not be adequately evaluated, as women represent a small proportion of personnel deployed to operational areas.

Similarly, a study comparing Indigenous and non-Indigenous Colombian populations identified ethnicity as a risk factor for asymptomatic malaria, with higher prevalence observed among individuals living in Indigenous communities—conditions associated with housing characteristics such as limited access to electricity, incomplete window and door screening, and proximity to forests and bodies of water. Ethnicity was also not significant, likely because soldiers do not reside in the environmental and housing conditions associated with increased risk in Indigenous communities [23].

A study conducted in Lao identified “any malaria case in the household” as a key predictor of asymptomatic malaria infection [46]. Although no statistically significant differences were detected here, a greater proportion of asymptomatic individuals (10%; 2/ 20) reported contact with symptomatic patients compared with healthy individuals (8.0%; 60/ 753) (Table 4).

Finally, key limitations include insufficient sampling in some municipalities, the cross-sectional design and excess of nominal variables, which restricts causal inference and potential biases. Future studies should incorporate larger and longitudinal designs with more quantitative variables to better characterize transmission dynamics, progression from asymptomatic to symptomatic infection, the variation in submicroscopic and subpatent malaria, the effectiveness of treatment and medical prognosis.

## Conclusion

In conclusion, the prevalence of asymptomatic malaria infection in Colombian military personnel was 2.59% in the departments of Antioquia, Chocó, Córdoba, and Nariño during 2022, as determined by conventional PCR and qPCR, with *P. falciparum* being the most prevalent species, followed by *P. vivax* and mixed infections. The prevalence of asymptomatic malaria by municipality, from highest to lowest, corresponded to El Bagre–Antioquia, Carepa–Antioquia, Tumaco–Nariño, Quibdó–Chocó, and Tierralta–Córdoba. Likewise, qPCR was identified as the most sensitive and cost-effective molecular method for diagnosing and genotyping asymptomatic malaria and may serve as a reference for developing surveillance guidelines for asymptomatic infection in the Colombian National Army. This would enable the timely treatment of asymptomatic individuals within Military Health Establishments, helping mitigate transmission foci in operational areas and thereby reducing malaria-associated morbidity and mortality among military personnel.

Finally, bed net use frequency, number of lifetime malaria episodes, number of malaria episodes in the last two years, department of origin, department of patrol, age, days of patrol, and number of patrol sites were identified as the main risk factors associated with asymptomatic malaria infection in Colombian military personnel. Consequently, the findings of this study support epidemiological recommendations to restructure malaria public health surveillance programs at national and international scales to incorporate the detection of asymptomatic *Plasmodium* spp. infections, enabling treatment of human reservoirs. Such efforts would contribute to reducing disease transmission and advancing malaria elimination in Colombia, across the region, and globally through governmental initiatives led by the National Institute of Health and the Ministry of Health and Social Protection, with transnational support from the Pan American Health Organization and the World Health Organization.

## Supporting information

S1 Table Primers and probes used for conventional PCR and qPCR detection of *Plasmodium* spp. Details of all primers and probes used for the genus-specific 18S rRNA qPCR assay with ERV-3 internal control, and for the multiplex species-specific qPCR assays for *P. falciparum*, *P. vivax*, and *P. malariae*, including sequence composition and fluorescent labeling.
(DOCX)

S2 Table Raw dataset of demographic, clinical, epidemiological, and laboratory variables for all study participants. Complete anonymized dataset used for all descriptive analyses, statistical comparisons, and molecular diagnostics. Variables include demographic characteristics, military deployment information, malaria history, protective measures, patrol activity, diagnostic test results (thick smear, peripheral smear, conventional PCR, nPCR, and qPCR), and sequencing outcomes.
(XLSX)
