# Supplementary material for: Prevalence and risk factors of asymptomatic Plasmodium spp. infection in the military population of the Colombian National Army
Source: PLoS Negl Trop Dis. 2026 Jul 2;20(7):e0014441. doi: 10.1371/journal.pntd.0014441 (PMC13349293; doi:10.1371/journal.pntd.0014441)
Supplement: S1 Table — Details of all primers and probes used for the genus-specific 18S rRNA qPCR assay with ERV-3 internal control, and for the multiplex species-specific qPCR assays for P. falciparum, P. vivax, and P. malariae, including sequence composition and fluorescent labeling. (DOCX) [file pntd.0014441.s001.docx]

**S1 Table. Primers and probes used for conventional PCR and qPCR detection of *Plasmodium* spp.** Details of all primers and probes used for the genus-specific 18S rRNA qPCR assay with ERV-3 internal control, and for the multiplex species-specific qPCR assays for *P. falciparum*, *P. vivax*, and *P. malariae*, including sequence composition and fluorescent labeling.

| **Assay** | **Target** | **Gene** | **Amplicon size (bp)** | **Oligonucleotide type** | **Primer/ Probe Name** | **Sequence (5'–3')** | **Fluorophore** | **Quencher** | **Reference** |
| --- | --- | --- | --- | --- | --- | --- | --- | --- | --- |
| Genus qPCR (screening) | *Plasmodium spp.* | 18S rRNA | 157–165 | Forward primer | Plasmo 1 | GTT AAG GGA GTG AAG ACG ATC AGA | – | – | [28] |
| Genus qPCR (screening) | *Plasmodium spp.* | 18S rRNA | 157–165 | Reverse primer | Plasmo 2 | AAC CCA AAG ACT TTG ATT TCT CAT AA | – | – | [28] |
| Genus qPCR (internal control) | Human (endogenous control) | ERV-3 | 135 | Forward primer | ERV-3F | ATG GGA AGC AAG GGA ACT AAT G | – | – | [29] |
| Genus qPCR (internal control) | Human (endogenous control) | ERV-3 | 135 | Reverse primer | ERV-3R | CCC AGC GAG CAA TAC AGA ATT T | – | – | [29] |
| Genus qPCR (screening) | *Plasmodium spp.* | 18S rRNA | 157–165 | Probe | Plaspro | ACC GTC GTA ATC TTA ACC ATA AAC TAT GCC GAC TAG | FAM | BHQ | [28] |
| Genus qPCR (internal control) | Human (endogenous control) | ERV-3 | 135 | Probe | ERV-3-probe | TCT TCC CTC GAA CCT GCA CCA TCA AGT CA | Cy5 | BHQ | [29] |
| Species qPCR (multiplex) | *P. falciparum* | 18S rRNA | – | Probe | Falcprobe | AGC AAT CTA AAA GTC ACC TCG AAA GAT GAC T | FAM | BHQ | [29] |
| Species qPCR (multiplex) | *P. vivax* | 18S rRNA | – | Probe | Vivprobe | AGC AAT CTA AGA ATA AAC TCC GAA GAG AAA ATT CT | HEX | BHQ | [29] |
| Species qPCR (multiplex) | *P. malariae* | 18S rRNA | – | Probe | Malaprobe | CTA TCT AAA AGA AAC ACT CAT | CAL FLUOR RED 610 | BHQ | [29] |
